# Perceived discrimination and refraining from seeking physician’s care in Sweden: an intersectional analysis of individual heterogeneity and discriminatory accuracy (AIHDA)

**DOI:** 10.1186/s12939-024-02291-4

**Published:** 2024-10-05

**Authors:** Mariam Hassan, Johan Öberg, Maria Wemrell, Raquel Perez Vicente, Martin Lindström, Juan Merlo

**Affiliations:** 1 Unit for Social Epidemiology, Department of Clinical Sciences, Faculty of Medicine, Lund University, Clinical Research Centre, Jan Waldenströms Street 35, Malmö, 214 28 Sweden; 2 Department of Health and Medical Care Management, Region Skåne Corporate Headquarter Office, Malmö, Sweden; 3 Department of Social Work, Faculty of Social Sciences, Linnaeus University, Kalmar, Sweden; 4 Centre for Primary Health Care Research, Region Skåne, Malmö, Sweden; 5 Department of Clinical Sciences, Faculty of Medicine, Social Medicine and Health Policy, Lund University, Malmö, Sweden

**Keywords:** Health inequities, Access to medical care, Perceived discrimination, Social determinants of health, Intersectionality, Sweden

## Abstract

**Background:**

Discrimination may further impede access to medical care for individuals in socially disadvantaged positions. Sociodemographic information and perceived discrimination intersect and define multiple contexts or strata that condition the risk of refraining from seeking physician’s care. By applying analysis of individual heterogeneity and discriminatory accuracy (AIHDA) we aimed to improve the mapping of risk by considering both strata average risk differences and the accuracy of such strata risks for distinguishing between individuals who did or did not refrain from seeking physician’s care.

**Methods:**

We analysed nine annual National Public Health Surveys (2004, 2007–2014) in Sweden including 73,815 participants. We investigated the risk of refraining from seeking physician’s care across 64 intersectional strata defined by sex, education, age, country of birth, and perceived discrimination. We calculated strata-specific prevalences and prevalence ratios (PR) with 95% confidence intervals (CI), and the area under the receiver operating characteristic curve (AUC) to evaluate the discriminatory accuracy (DA).

**Results:**

Discriminated foreign-born women aged 35–49 with a low educational level show a six times higher risk (PR = 6.07, 95% CI 5.05–7.30) than non-discriminated native men with a high educational level aged 35–49. However, the DA of the intersectional strata was small (AUC = 0.64). Overall, discrimination increased the absolute risk of refraining from seeking physician’s care, over and above age, sex, and educational level.

**Conclusions:**

AIHDA disclosed complex intersectional inequalities in the average risk of refraining from seeking physician’s care. This risk was rather high in some strata, which is relevant from an individual perspective. However, from a population perspective, the low DA of the intersectional strata suggests that potential interventions to reduce such inequalities should be universal but tailored to the specific contextual characteristics of the strata. Discrimination impairs access to healthcare.

**Supplementary Information:**

The online version contains supplementary material available at 10.1186/s12939-024-02291-4.

## Introduction

In Sweden, Swedish law enforces equal access to medical care based on needs [1], aligning with the core aim of universal healthcare coverage in the Swedish public health policy [1, 2]. According to the principle of equity, health disparities should be prevented and eliminated, underscoring the crucial role of equitable access to medical care for effective and efficient high-quality care [3]. Achieving equity in medical care requires political decisions ideally grounded on the best available information and state-of-the-art epidemiological analyses. This necessitates comprehensive analyses considering factors known to condition unequal access to healthcare including socioeconomic positions [4], gender/sex [5], race/ethnicity/ racialisation [6], migration status [7], including language barriers, and age [8], as well as transgender identity [9, 10], religion [11], disability [12], and sexual orientation [13]. Considering Sweden’s increasing demographic diversity in 2023, with 20% of the population born in another country than Sweden, predominantly from Asia, Europe, and Africa [14], it is crucial to examine factors impacting equal access to healthcare. Social discrimination, the different or unfair treatment of individuals from a socially defined group based on socially derived beliefs about their group, rooted in patterns of dominance and oppression [15], impacts psychosocial functioning. This may limit individuals’ control, influence and societal participation – critical elements for ensuring access to services such as medical care and promoting equal health [16–18]. Social discrimination, and its impact on health, is thus a growing public health concern that contributes to explaining population patterns of health inequities [19–21]. However, the impact of discrimination and experiences of discrimination on aspects of healthcare are still insufficiently investigated [22].

Previous quantitative research has provided ample evidence of the existence of socioeconomic health inequalities. However, it has been criticized for typically focusing on one or a few separated socioeconomic variables, disregarding complex inequities across multiple interlocked social dimensions [23–25]. Intersectionality is a theoretical framework, originally termed by Kimberlé Crenshaw [23], emphasizing how multiple social identities, such as gender, class, and race/ethnicity/racialization can intersect and interact with each other [23] defining multiple contexts of privilege, oppression and disadvantage [24, 25]. The inclusion of an intersectional theoretical framework has been promoted in quantitative population health research [26–28], to provide improved mapping of inequalities in health and better elucidate patterns of disadvantage (e.g., [22, 28–30]).

Furthermore, previous epidemiological health inequalities studies may have oversimplified group differences by attributing the same average value to all individuals in the group, a phenomenon denominated as the “tyranny of the averages” [27]. This approach disregards individual heterogeneity around group averages and overlaps of risk between groups [31, 32], potentially leading to unnecessary stigmatization of individuals deemed “high-risk”, and giving false expectations to individuals in the “low-risk” groups [26, 31, 32]. The heterogeneity referred to here is the differences in the tendency to refrain from seeking physician’s care (PC). *Analyses of individual heterogeneity and discriminatory accurac*y (AIHDA), including using random effects multilevel models and then denominated as MAIHDA [33], are used in quantitative intersectional epidemiology [26, 28, 30] as a response, and consider intersectional groups as contexts. (M)AIHDA emphasizes assessing discriminatory accuracy (DA) in addition to group averages when studying multiple groups. This approach evaluates differences between group averages as well as the capacity of strata to accurately classify individuals based on the outcome of interest, which might mitigate the “tyranny of the averages” noted above [31]. Intersectional (M)AIHDA provides an improved methodological framework for the study of healthcare inequalities.

In the present study, we apply intersectional AIHDA to acquire a better understanding of how the risk of refraining from seeking PC when perceiving oneself to need care is distributed across intersectional strata defined by combinations of self-reported experiences of injustice (i.e., practices that benefit certain individuals or groups at the expense of others, perpetuating oppression, discrimination and inequality) in this study defined as individuals reporting offensive or abusive treatment due to ethnicity, sex/gender, sexual orientation, age, religion or disability and several socioeconomic and demographic dimensions. When doing so, we focus particularly on the risk attributable to injustice.

## Population and methods

### Study population

The study’s data was obtained from nine National Public Health Surveys (NPHS) executed in Sweden during 2004, 2007–2014 [34]. The surveys cover questions on health, lifestyle, and living conditions. From 2004 to 2016 the surveys were executed annually with a random selection comprising 20,000 individuals aged 16–84 [35]. However, since 2018 the survey has been conducted biannually, and the sample has increased to contain 40,000 individuals [35]. The response rate ranges from 60.8% in 2004 to 42.1% in 2018 [35].

**Fig. 1:**
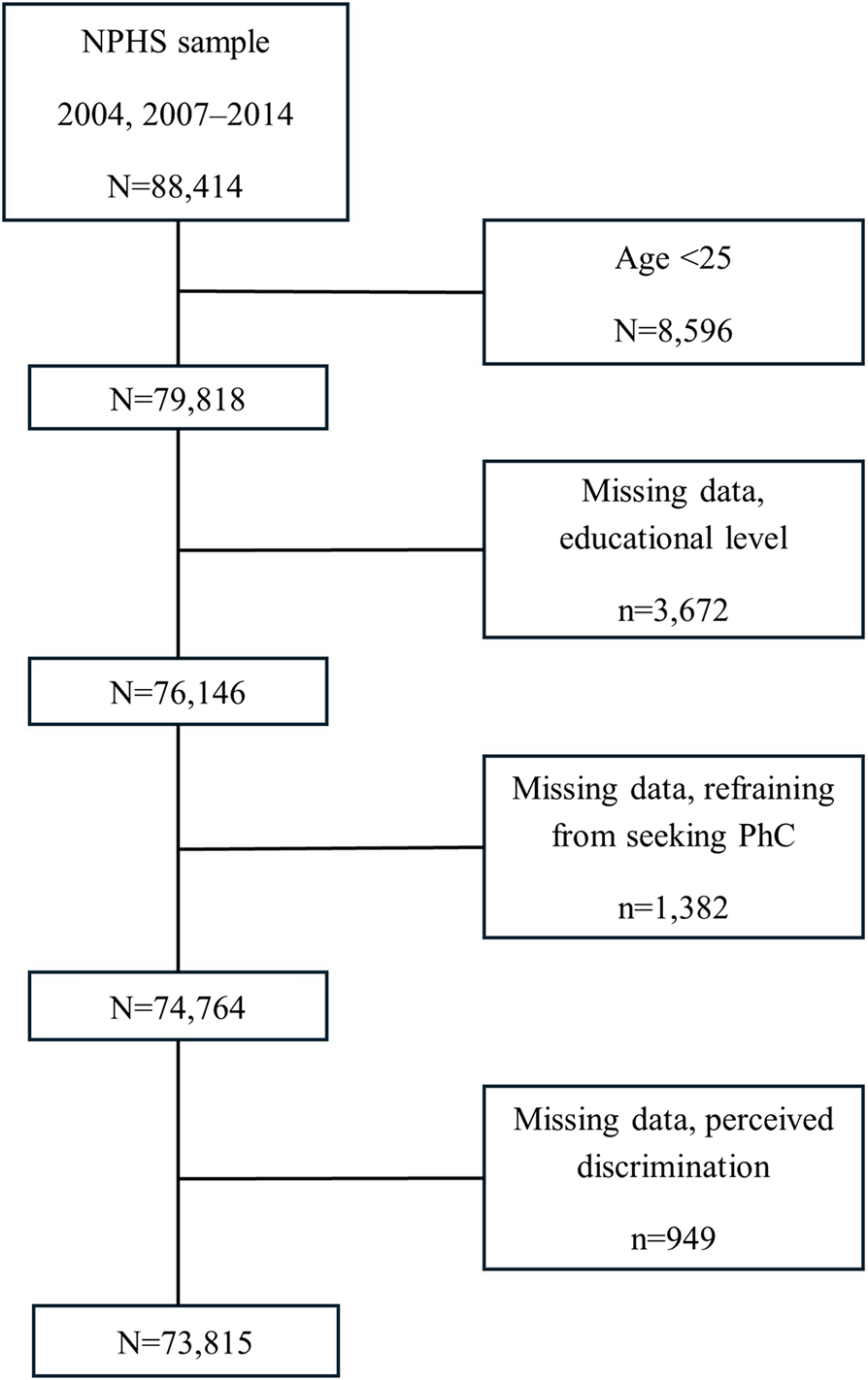
Study population flowchart. From the original population consisting of data from nine National Public Health Surveys (2004, 2007–2014), we excluded respondents younger than 25 and missing data on educational level, refraining from seeking physician's care (PC), and perceived discrimination, obtaining a final sample of 73,815 respondents

The sample in this study consists of pooled data including 88,414 respondents to the NPHS from 2004, 2007–2014. The question serving as the outcome of this study was removed from the questionnaire in 2005–2006 and 2015 and onward, and thus surveys from these respective years (2005–2006, 2015–) were excluded from this study. Participants younger than 25, i.e., 16–24-year-olds were excluded, as education is used as an indicator of socioeconomic position and only individuals aged 25 and older were deemed to have had the time to attain a higher education. This exclusion was made to avoid bias when categorizing individuals according to their socioeconomic position. Finally, after excluding individuals with missing data the study population comprised 73,815 individuals aged 25–84 years (Fig. 1).

### Data collection

The data was provided by the Swedish Public Health Agency (PHA) and it was obtained through postal and online self-administered questionnaires executed in cooperation between PHA, Statistics Sweden, and the Swedish Association of Local Authorities and Regions [35, 36]. The questionnaires were available in several languages, namely Swedish, English (years 2005, 2007, 2012, 2014) and Finnish (years 2005, 2006, 2010, 2014). The PHA is responsible for the translations and has translated the survey internally by employees with language expertise, hiring external consultants and by the PHA’s public procurement of translation services. The study sample was randomly collected from the Total Population Register (TPR). The NPHS database contains updated individual and household information on socioeconomic variables obtained by record linkage with population registers administered by Statistics Sweden using a unique personal identification number. During the collection of the sample, it was possible to identify people who no longer belonged to the population due to emigration or being deceased. For more detailed information on the process of inclusion, exclusion, and missing data, we refer to the NPHS reports [36]. Generally, men, individuals born in another country than Sweden (except for years 2008, 2012–3 where individuals born in other Nordic countries than Sweden responded to a similar extent), individuals with lower education levels, lower income, and younger individuals responded to a lesser extent (e.g., [36]). Statistics Sweden created weights to compensate for the non-proportional demographical structure of the sample [36]. However, it cannot be excluded that information bias due to missing data (non-participation) may reduce the external validity of the results [36, 37].

### Assessment of variables

#### Outcome variable – refraining from seeking physician’s care (PC)

The outcome variable was coded into a dummy variable *“refraining from seeking medical care”* (yes/no) and was based on the question *“Have you during the last three months considered yourself in need of medical care but refrained from seeking care?”*. In the English version of the questionnaire the Swedish term “*läkarvård*” was translated as *“medical care”*. However, the Swedish term mainly refers to care attained by a physician whereas *“medical care”* in English could refer to the provision of care by any healthcare professional. Thus, the minority of participants who responded to the survey in English may have had a different interpretation of the question. Therefore, in the rest of the paper, we use the term “physician’s care” (PC) as it better expresses the Swedish term *“läkarvård”.*

#### Perceived discrimination

Perceived discrimination was measured through questions about offensive or abusive treatment resulting in feelings of humiliation. The questions included various perceived reasons for discrimination, as well as the frequency of discrimination. The frequency of perceived discrimination was based on the question *“During the last three months, have you been treated in a way that made you feel humiliated?”*. The response options were *“yes, sometimes”*, *“yes, several times”* and *“no”*. Participants who reported having experienced any form of humiliation were asked to give one or more reasons that were attributed to this offensive or abusive treatment. The response options included ethnicity, sex/gender, sexual orientation, age, disability, religion, gender identity, appearance, sexual identity, skin colour, other, and do not know. These grounds varied across the survey years, except for ethnicity, sex/gender, sexual orientation, age, disability, religion, other, and do not know. Therefore, we decided to base the variable perceived discrimination on the aforementioned grounds consistent throughout the survey years, and the individuals responding “other” or “do not know” were considered non-discriminated. The grounds were dichotomized as discrimination (yes vs. no). In the year 2004, questions were also posed about where or by who the respondent was subjected to humiliating treatment (e.g., healthcare, public employment service, or close relatives), however, this information is not used in this study due to only being available for one year. In the rest of the study, we use the terms “discriminated” and “discrimination” synonymously with the perceived phenomena.

#### Socioeconomic and demographic variables

Official information on sex/gender was obtained from the population register and coded as *“sex”* (woman/man). Age was classified into four different groups, namely 25–34, 35–49, 50–64, and 65–84 years. Educational level was dichotomized (high vs. low), where the secondary level or lower (12 years of education or less) was considered as “low” and the postsecondary level as “high”. *Country of birth* was dichotomized (native vs. foreign-born), with respondents born in Sweden categorized as native and those born in another country as foreign-born.

#### The multicategorical intersectional variable

For the purpose of the intersectional analysis, we created a multicategorical variable by not considering just one category at a time but the intersections of categories, combining the two categories of each of the following variables respectively, discrimination, sex, educational level and country of birth, and the four categories of age. Hence, we defined 64 intersectional strata (i.e., 2 × 2 × 2 × 2 × 4 = 64). The reference stratum used in the analysis was non-discriminated native men aged 35–49 with a high educational level. This stratum was *a priori* assumed to have the highest structural privilege and, thus, the lowest risk for refraining from seeking PC. This notion was based on previous literature [25, 38].

### Epidemiological and statistical analyses

#### Purpose of analysis and prevalence estimation

We applied intersectional AIHDA as described previously [26, 39]. The analysis had two main purposes, *(i)* to provide an intersectional mapping of the distribution of the prevalence or absolute risk (AR) of refraining from seeking PC across the 64 intersectional strata, and *(ii)* to inform on the DA of the statistical model. That is, do the average risks of the intersectional strata accurately classify the individuals according to their refrainment from seeking PC?

In the analysis, we expanded the dataset using the survey weights provided by SCB. For each intersectional stratum, we calculated the prevalence, absolute risk (AR) or positive predictive value (PPV) with a 95% confidence interval (CI) of the dichotomized variable refraining from seeking PC. Furthermore, for each pair of strata differing only on experiences of discrimination, we calculated the absolute risk difference or attributable risk due to discrimination (ARD) and 95% CI. We also performed the analyses applying the survey weights provided by Statistics Sweden.

#### Intersectional analysis of individual heterogeneity and discriminatory accuracy

We performed eight consecutive regression models. Since the prevalence of refraining from seeking PC was rather high, odds ratios would not represent good estimations of the relative risks, and we therefore did not use logistic regression. Instead, we used Cox Proportional Hazard Regression with a constant follow-up time equal to 1, to obtain prevalence ratios (PR) with 95% CI [40]. The first model (Table 2, Model 1) included only the survey years as the independent variable. The second model (Model 2) expanded on Model 1 by adding one new variable at a time. Model *2a* included age, Model *2b* included sex, Model *2c* included country of birth, Model *2d* included educational level, and Model *2e* included discrimination. In the third main effects model (Model 3) all variables were entered as separate dimensions. The fourth model (Model 4) was like Model 3, but the information was included using the multicategorical intersectional variable.

After the regression analysis was conducted, in each model, we computed the predicted probability of refraining from seeking PC and used it to obtain the receiver operating characteristics (ROC) curve and the area under the ROC curve (AUC) [41, 42]. The ROC curve plots the true positive fraction (sensitivity) against the false positive fraction (1-specificity) across thresholds of predicted probability of refraining from seeking PC. The AUC is a measure of DA, and it informs on the capacity of a model to correctly discriminate between individuals who refrain from seeking PC from individuals who do not. The larger the AUC the larger the DA. The AUC can take a value between 0.5 and 1, with 1 representing perfect discriminative accuracy and 0.5 indicating no discriminative accuracy at all. Using the criteria proposed by Hosmer and Lemeshow [43], we classified the DA as absent or very small (0.50 ≤ AUC ≤ 0.60), small (0.60 < AUC ≤ 0.70), large (0.70 < AUC ≤ 0.80), or very large (AUC > 0.80). We compared the different AUCs to evaluate the DA of the models. In the presence of multiplicative interaction of effects, the AUC of Model 4 would be higher than in Model 3.

## Results

Overall, the AR, prevalence or PPV of having refrained from seeking medical care in the pooled survey database was 16.37%. This figure was rather similar in each year-specific survey database except for a peak (21%) in 2007 (Table 1). The AR of refraining from seeking PC decreased with age, being twice as high in the youngest group compared to the oldest group. The AR was higher among women, foreign-born and individuals with a low educational level as compared to men, natives, and individuals with a high educational level, respectively. The AR of refraining from seeking PC was almost three times higher among those reporting discrimination (40.72%), compared to those who did not (14.98%).

**Table 1:** Weighted and unweighted descriptive statistics and prevalence (P) of refraining from seeking physician's care according to the Swedish National Public Health Survey from 2004, 2007–2014 by categories of age, sex, country of birth, educational level and perceived discrimination

	**Refraining from seeking physician's care**			
	Unweighted sample (*N*= 73,815)		Weighted sample (*N*= 5,880,633)	
	N (%)	P (95% CI)	N (%)	*P* (95% CI)
**Age (years)**				
25-34	10460 (14.17%)	19.51% (18.76–20.28)	1047088 (17.81%)	20.41% (19.58–21.26)
35-49	20301 (27.50%)	16.99% (16.48–17.52)	1826683 (31.06%)	18.12% (17.54–18.72)
50-64	23697 (32.10%)	14.63% (14.19–15.09)	1802473 (30.65%)	15.88% (15.37–16.41)
65-84	19357 (26.22%)	10.17% (9.75–10.60)	1204389 (20.48%)	10.91% (10.44–11.41)
**Sex**				
Men	33715 (45.67%)	13.29% (12.93–13.65)	2954710 (50.24%)	14.96% (14.53–15.40)
Woman	40100 (54.33%)	16.07% (15.72–16.44)	2925923 (49.76%)	17.78% (17.37–18.21)
**Country of birth**				
Native	64837 (87.84%)	24.88% (24.00–25.79)	4913110 (83.55%)	14.45% (14.15–14.75)
Foreign-born	8978 (12.16%)	13.41% (13.15–13.67)	967523 (16.45%)	26.09% (25.11–27.10)
**Educational level**				
High	29178 (39.53%)	13.65% (13.26–14.05)	2098122 (35.68%)	15.15% (14.68–15.63)
Low	44637 (60.47%)	15.55% (15.22–15.89)	3782511 (64.32%)	17.04% (16.66–17.44)
**Perceived discrimination**				
Yes	3637 (4.93%)	37.89% (36.33–39.48)	316544 (5,38%)	40.72 % (38.97–42.50)
No	70178 (95.07%)	13.61% (13.35–13.86)	5564089 (94.62%)	14.98% (14.68–15.28)
**Survey year**				
2004	9619 (13.03%)	13.64% (12.97–14.34)	593466 (10.09%)	14.40% (13.67–15.16)
2007	4505 (6.10%)	19.98% (18.84–21.17)	610958 (10.39%)	21.00% (19.74–22.32)
2008	8768 (11.88%)	14.87% (14.14–15.63)	616518 (10.48%)	15.80% (15.00–16.64)
2009	8223 (11.14%)	14.65% (13.91–15.44)	623814 (10.61%)	16.24% (15.36–17.15)
2010	8655 (11.73%)	14.64% (13.91–15.40)	672658 (11.44%)	15.90% (15.07–16.77)
2011	8523 (11.55%)	14.99% (14.25–15.77)	683222 (11.62%)	16.33% (15.48–17.22)
2012	8693 (11.78%)	14.44% (13.71–15.19)	694519 (11.81%)	16.21% (15.35–17.10)
2013	8508 (11.53%)	15.17% (14.43–15.95)	693022 (11.78%)	16.54% (15.69–17.43)
2014	8321 (11.27%)	13.39% (12.67–14.14)	692456 (11.78%)	15.05% (14.20–15.95)

**Fig. 2:**
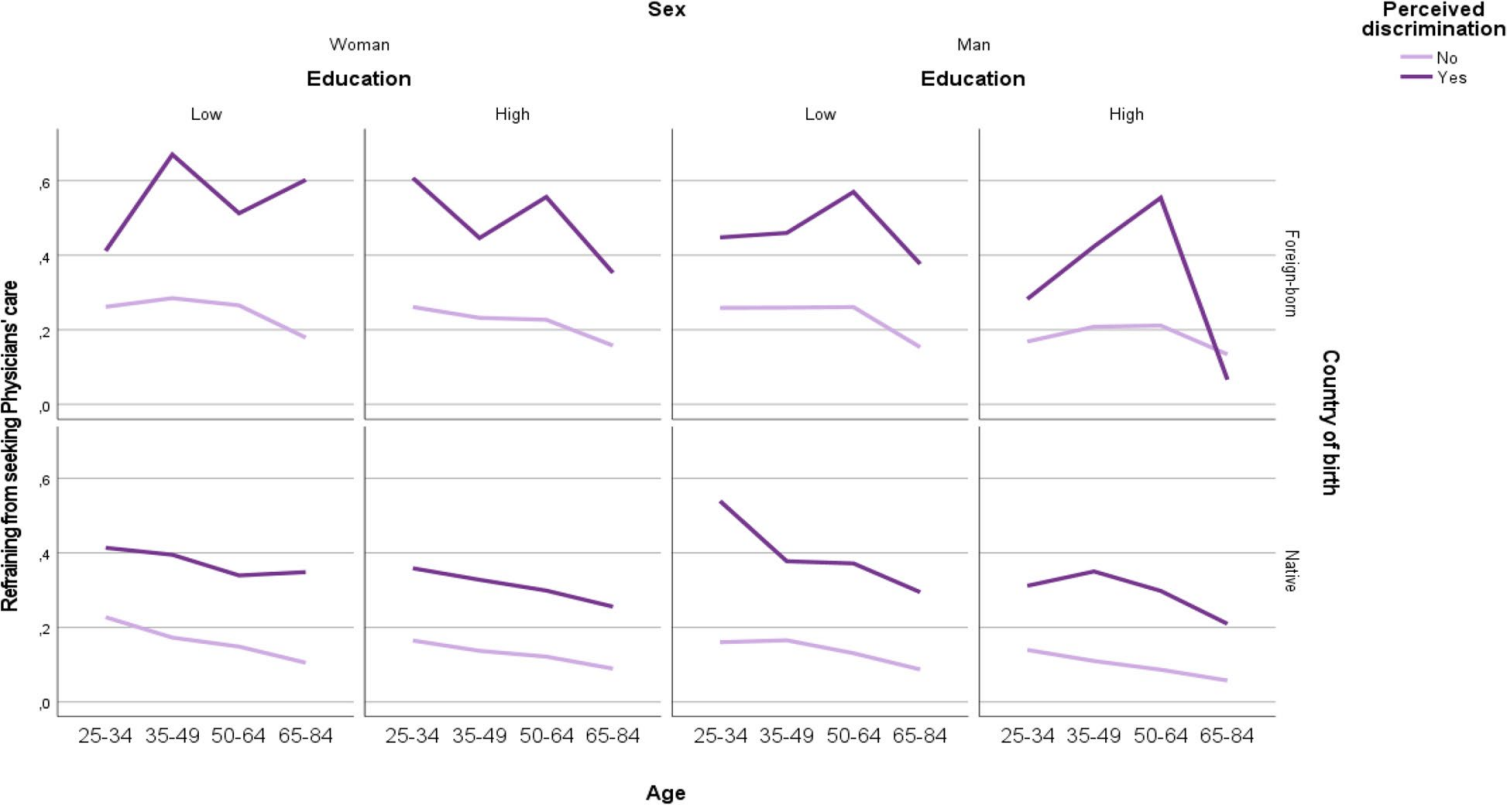
Prevalence of refraining from seeking PC in the 64 intersectional strata according to the Swedish National Public Health Survey (NPHS) from 2004, 2007–2014

Figure 2 shows the prevalence or AR of refraining from seeking PC in the 64 intersectional strata (see also supplementary material S1 for information on the number of individuals, and the AR and PRs of each stratum). Here we see a clear pattern of risk as, overall, the prevalence of refraining from seeking PC was higher among discriminated individuals compared to those who were not, across all the different categorisations of country of birth, educational level, sex, and age. While the prevalence of refraining from seeking PC differed depending on age, sex and education among native and foreign-born individuals, foreign-born men and women with experiences of discrimination have a high risk of refraining from seeking PC across all categories of age and educational level.

About 67% of the discriminated 35–49-year-old foreign-born women with a low educational level had refrained from seeking PC, while this number was much lower in the same group although without experiencing discrimination (28%). This figure was almost 57% among discriminated foreign-born men aged 50–64 with a low educational level. However, the prevalence was 10 times lower (i.e., 5.8%) among non-discriminated 65–84 -year-old native men with a high educational level and almost only 9% among non-discriminated native women aged 65–84 with a high educational level.

Table 2 shows the PRs of refraining from seeking PC obtained in the eight consecutive regression models. The PRs obtained from the simple regressions in models 2a–e provide similar information to that obtained in Table 1. For instance, in Model 2e, the relative risk of refraining from seeking PC was higher among those reporting discrimination compared to those who did not (PR = 2.72), and Model 2c shows a higher relative risk among foreign-born individuals compared to natives (PR = 1.81). In the multiple regression (Model 3), the mutually adjusted PRs of age groups 25–34 and 35–49, sex, country of birth and discrimination became somewhat reduced. However, the PR of educational level showed an increase while the PR of the age group 50–64 remained unchanged. Table 2, also informs that the DA of Model 1 including only the variable survey years had a “very small” DA (AUC = 0.53). The DA of Models 2a–e was also “very small”. The inclusion of all the variables in Model 3 only slightly increased the DA (AUC = 0.64). Compared to Model 3, the DA did not change when modelling the multicategorical variable in Model 4, indicating an absence of multiplicative interaction of effects.

**Table 2:** Prevalence ratios (PR) of refraining from seeking PC with 95% confidence intervals (CI) obtained in eight consecutive regression models using weighted data from the Swedish National Public Health Surveys 2004, 2007–2014

	**Model 1**	**Model *2a***	**Model *2b***	**Model *2c***	**Model *2d***	**Model *2e***	**Model 3**	**Model 4**^**a**^
		PR (95% CI)	PR (95% CI)	PR (95% CI)	PR (95% CI)	PR (95% CI)	PR (95% CI)	
**Age (years)**								
** 25–34**		1.86 (1.75–1.98)					1.82 (1.71–1.93)	
** 35–49**		1.66 (1.57–1.75)					1.65 (1.56–1.74)	
** 50–64**		1.45 (1.37–1.53)					1.45 (1.37–1.53)	
** 65–84**		Reference					Reference	
**Sex**								
** Man**			Reference				Reference	
** Woman**			1.19 (1.15–1.24)				1.16 (1.12–1.21)	
**Country of birth**								
** Native**				Reference			Reference	
** Foreign-born**				1.81 (1.73–1.89)			1.65 (1.58–1.72)	
**Educational level**								
** High**					Reference		Reference	
** Low**					1.13 (1.08–1.17)		1.28 (1.23–1.33)	
**Perceived discrimination**								
** Yes**						2.72 (2.59–2.85)	2.33 (2.22–2.45)	
** No**						Reference	Reference	
**Survey year**								
** 2004**	Reference	Reference	Reference	Reference	Reference	Reference	Reference	Reference
** 2007**	1.46 (1.35–1.58)	1.46 (1.35–1.58)	1.46 (1.35–1.58)	1.46 (1.34–1.58)	1.47 (1.35–1.59)	1.47 (1.36–1.59)	1.49 (1.38–1.61)	1.49 (1.37–1.61)
** 2008**	1.10 (1.02–1.18)	1.10 (1.03–1.19)	1.10 (1.02–1.18)	1.09 (1.01–1.17)	1.10 (1.03–1.19)	1.12 (1.04–1.21)	1.14 (1.06–1.22)	1.13 (1.05–1.21)
** 2009**	1.13 (1.05–1.22)	1.14 (1.06–1.23)	1.13 (1.05–1.22)	1.11 (1.03–1.19)	1.14 (1.05–1.22)	1.15 (1.07–1.24)	1.16 (1.08–1.25)	1.16 (1.07–1.24)
** 2010**	1.10 (1.03–1.19)	1.15 (1.06–1.23)	1.10 (1.03–1.19)	1.09 (1.01–1.17)	1.11 (1.03–1.20)	1.12 (1.04–1.20)	1.16 (1.08–1.25)	1.16 (1.07–1.24)
** 2011**	1.13 (1.05–1.22)	1.18 (1.10–1.27)	1.13 (1.05–1.22)	1.11 (1.03–1.20)	1.14 (1.06–1.23)	1.15 (1.07–1.24)	1.20 (1.11–1.29)	1.19 (1.10–1.28)
** 2012**	1.13 (1.04–1.21)	1.17 (1.09–1.26)	1.13 (1.04–1.21)	1.10 (1.02–1.19)	1.13 (1.05–1.22)	1.15 (1.07–1.24)	1.19 (1.11–1.28)	1.19 (1.10–1.28)
** 2013**	1.15 (1.07–1.24)	1.20 (1.12–1.29)	1.15 (1.07–1.24)	1.13 (1.05–1.21)	1.16 (1.08–1.25)	1.16 (1.08–1.25)	1.22 (1.13–1.31)	1.21 (1.12–1.30)
** 2014**	1.05 (0.97–1.13)	1.09 (1.01–1.18)	1.05 (0.97–1.13)	1.02 (0.95–1.11)	1.05 (0.98–1.14)	1.06 (0.98–1.14)	1.11 (1.03–1.20)	1.10 (1.02–1.19)
***Discriminatory Accuracy***								
** AUC**	0.52 (0.52–0.53)	0.58 (0.57–0.58)	0.54 (0.54–0.55)	0.57 (0.56–0.57)	0.53 (0.53–0.54)	0.56 (0.56–0.57)	0.64 (0.63–0.64)	0.64 (0.63–0.64)

^a^See Table 3 and supplementary material 1 for extended information on Model 4. AUC: area under the receiver operating characteristics curve

Table 3 shows the 10 strata with the highest and the 10 strata with the lowest PRs of refraining from seeking PC, compared with the reference stratum (i.e., non-discriminated native men aged 35–49 with high educational level). All 10 strata with the highest relative risk included discriminated individuals. Only one high-risk stratum included native-born individuals, and only one stratum included individuals from the oldest age group. On the other hand, 9 out of the 10 strata with the lowest relative risk included non-discriminated individuals, and 8 strata included native individuals. Furthermore, seven strata included highly educated individuals, and none of the strata comprised respondents from the age group 25–34. The strata with the lowest risk, PR = 0.52, included non-discriminated native men aged 65–84 with a high educational level.

**Table 3:** Extended information on model 4 from Table 2. Weighted statistic regression analysis including the intersectional multicategorical variable and using as reference the stratum of non-discriminated highly educated native men aged 35–49. The table shows only the 10 strata with the highest and the 10 with the lowest risk of refraining from seeking PC. Values are prevalence ratios (PR) and 95% confidence intervals (CI) from the Swedish National Public Health Surveys 2004, 2007–2014. Complete information on all strata is available in supplementary material 1

**Rank**	Perceived discrmination		Country of birth	Sex	Educational level		Age	PR (95% CI)
**The 10 strata with the lowest risk**								
1	No	Native		Men		High	65–84	0.52 (0.42–0.65)
2	Yes	Foreign-born		Men		High	65–84	0.60 (0.08–4.28)
3	No	Native		Men		High	50–64	0.79 (0.67–0.92)
4	No	Native		Men		Low	65–84	0.80 (0.69–0.91)
5	No	Native		Women		High	65–84	0.82 (0.68–0.98)
6	No	Native		Women		Low	65–84	0.96 (0.84–1.10)
Reference	*No*	*Native*		*Men*		*High*	*35–49*	*1.0*
8	No	Native		Women		High	50–64	1.11 (0.96–1.27)
9	No	Native		Men		Low	50–64	1.19 (1.05–1.35)
10	No	Foreign-born		Man		High	65–84	1.20 (0.80–1.79)
**The 10 strata with the highest risk**								
55	Yes	Foreign-born		Women		High	35–49	4.08 (3.14–5.30)
56	Yes	Foreign-born		Men		Low	35–49	4.21 (3.13–5.65)
57	Yes	Foreign-born		Women		Low	50–64	4.63 (3.58–5.98)
58	Yes	Native		Men		Low	25–34	4.90 (3.95–6.09)
59	Yes	Foreign-born		Men		High	50–64	4.92 (3.64–6.66)
60	Yes	Foreign-born		Women		High	50–64	4.96 (3.90–6.31)
61	Yes	Foreign-born		Men		Low	50–64	5.20 (4.10–6.59)
62	Yes	Foreign-born		Women		High	25–34	5.37 (4.25–6.78)
63	Yes	Foreign-born		Women		Low	65–84	5.45 (4.28–6.95)
64	Yes	Foreign-born		Women		Low	35–49	6.07 (5.05–7.30)
**Discriminatory Accuracy**								
**AUC**								0.64 (0.63–0.64)

*AUC* Area under the receiver operating characteristics curve

Table 4 shows the ARs of refraining from seeking PC in pairs of similar strata differing only on reported discrimination. It also shows the ARD attributable to discrimination for each strata pair. The ARD informs on both the association between discrimination and refraining from seeking PC and the excess risk that could be eliminated by preventing discrimination in a specific stratum. The strata with the highest ARD = 42.32 was the pair composed of foreign-born women with low educational level aged 65–84 years. The lowest ARD = -6.74 was identified between the pair of strata composed of foreign-born men with high educational level aged 65–84 years. This was also the only pair where the non-discriminated individuals had a higher risk compared to the discriminated group. This pair together with three other pairs showed insignificant differences. The lowest ARD = 15.13 following the aforementioned group was between the strata composed of 65–84-year-old native men with a high educational level. As many as 19 strata pairs had an ARD ≥ 20, and in 12 pairs the ARD was between 11.42 and 19.53, although three of these pairs ARD were conclusive according to the 95% CI. Seven out of the 10 strata with the highest ARDs included foreign-born individuals and individuals with low educational level. Six out of those strata included women.

**Table 4:** Prevalence or absolute risk (AR) of refraining from seeking physician's care and absolute risk difference (ARD) attributable to perceived discrimination with 95% confidence interval (CI) among pairs of otherwise similar intersectional strata regarding age, sex, educational achievement and country of birth (COB). Analysis performed using survey weights

**Age**	Sex	Education	COB	Discrimination		
				**No**	**Yes**	**Yes vs. No**
				AR (95% CI)	AR (95% CI)	ARD (95% CI)
25–34	Women	Low	Foreign born	26.16 (20.99–32.08)	41.14 (28.05–55.62)	14.98 (-0.21–30.18)
		High		26.11 (21.67–31.10)	60.71 (47.69–72.37)	34.60 (21.16–48.05)
		Low	Native	22.77 (20.79–24.88)	41.38 (34.81–48.26)	18.61 (11.54–25.67)
		High		16.51 (15.06–18.07)	35.89 (31.23–40.83)	19.38 (14.34–24.42)
	Men	Low	Foreign born	25.87 (20.57–31.99)	44.78 (30.74–59.71)	18.91 (2.94–34.88)
		High		16.84 (12.52–22.28)	28.26 (13.74–49.33)	11.42 (-7.56–30.40)
		Low	Native	16.06 (14.37–17.91)	53.91 (43.84–63.67)	37.85 (27.65–48.05)
		High		13.99 (12.41–15.74)	31.20 (20.36–44.59)	17.21 (4.79–29.63)
35–49	Women	Low	Foreign born	28.49 (25.10–32.13)	66.98 (56.07–76.33)	38.49 (27.66–49.33)
		High		23.20 (20.07–26.65)	44.61 (34.55–55.13)	21.40 (10.46–32.35)
		Low	Native	17.30 (16.15–18.51)	39.53 (33.30–46.11)	22.23 (15.68–28.77)
		High		13.73 (12.71–14.82)	32.80 (27.26–38.85)	19.06 (13.15–24.97)
	Men	Low	Foreign born	25.94 (22.29–29.96)	45.98 (34.40–58.01)	20.04 (7.41–32.66)
		High		20.83 (17.38–24.75)	42.33 (30.71–54.86)	21.50 (8.65–34.35)
		Low	Native	16.57 (15.43–17.78)	37.79 (29.15–47.28)	21.22 (11.98–30.46)
		High		11.02 (9.88–12.27)	35.02 (24.09–47.80)	24.00 (11.89–36.12)
50–64	Women	Low	Foreign born	26.55 (23.56–29.78)	51.27 (39.30–63.10)	24.72 (12.19–37.24)
		High		22.70 (19.03–26.85)	55.61 (43.79–66.82)	32.91 (20.55–45.27)
		Low	Native	14.88 (13.99–15.81)	33.96 (28.15–40.30)	19.09 (12.92–25.25)
		High		12.19 (11.16–13.31)	29.90 (23.73–36.90)	17.71 (11.02–24.41)
	Men	Low	Foreign born	26.10 (22.92–29.55)	56.98 (44.65–68.50)	30.88 (18.28–43.48)
		High		21.14 (17.09–25.86)	55.38 (39.97–69.83)	34.24 (18.23–50.25)
		Low	Native	13.11 (12.23–14.03)	37.20 (30.12–44.88)	24.10 (16.61–31.58)
		High		8.69 (7.70–9.80)	29.83 (20.76–40.83)	21.14 (10.95–31.34)
65–84	Women	Low	Foreign born	17.90 (15.15–21.03)	60.22 (46.38–72.59)	42.32 (28.60–56.04)
		High		15.78 (11.16–21.83)	35.30 (17.24–58.83)	19.53 (-3.09–42.15)
		Low	Native	10.55 (9.79–11.37)	34.86 (27.58–42.92)	24.31 (16.54–32.07)
		High		8.96 (7.77–10.31)	25.53 (18.17–34.61)	16.57 (8.22–24.92)
	Men	Low	Foreign born	15.32 (12.46–18.70)	37.68 (22.56–55.64)	22.36 (4.94–39.78)
		High		13.42 (0.02–19.50)	6.67 (0.90–36.13)	-6.74 (-20.63–7.15)
		Low	Native	8.75 (8.03–9.52)	29.48 (22.18–38.01)	20.73 (12.73–28.73)
		High		5.78 (4.79–6.97)	20.91 (12.19–33.50)	15.13 (4.42–25.84)

## Discussion

In the present study, we investigated refraining from seeking physician’s care in Sweden, by adopting an intersectional AIHDA. Our study corroborates previous research findings by showing socioeconomic and demographic inequalities in access to medical care [8, 44]. However, previous studies have primarily analysed one or a few socioeconomic variables at a time and focused on differences between group averages. Our study fills those gaps and adds to existing knowledge in several ways. Firstly, by using an intersectional matrix we provide a detailed mapping of how the average risk is distributed across many different intersectional strata. For instance, the absolute risk of PPV in some strata was rather high so from an individual perspective, being a discriminated 35–49-year-old foreign-born woman with a low educational level conveys a high risk of refraining from seeking physician’s care. Secondly, by including measures of DA we also consider the individual heterogeneity around the strata’s averages and the overlapping of risk between strata. From a population perspective, the small AUC indicates that most people refraining from seeking physician’s care exist in the strata with lower risk. Considering the individual and the population perspectives simultaneously we can avoid unnecessary stigmatization of the individuals in the high-risk strata, prevent false expectations among the individuals in the low-risk strata, as well as provide a better basis for informed decisions, leading to more effective and efficient interventions.

Applying intersectional AIHDA analysis, we found that, overall, the prevalence of refraining from seeking PC in Sweden was 16% which is lower compared to other studies conducted relatively recently in Sweden [8, 45]. However, our results indicate the existence of complex inequalities affecting the risk of refraining from seeking PC and revealing population patterns in the distribution of deprivation and privilege. All the strata except for one stratum comprising individuals reporting discrimination had a higher AR compared to the strata including individuals of the same age, sex, educational level, and country of birth, reporting no discrimination. The PR showed similar patterns. Patterns discerned from the ARDs and PRs show that suffering from discrimination, being foreign-born and, to some extent, having a low educational level were dimensions most strongly associated with refraining from seeking PC. Nevertheless, we found some exceptions in the patterns of the ARD and PRs indicating that different social dimensions are interlocked and subject to complex influences, rather than being independent [25] and, thereby, potentially modifiable. This implies that the relationships among social dimensions can be influenced or altered, as they depend on various factors. While an intersectional framework helps us discern how social identities intersect and contribute to particular experiences, other theoretical approaches can be used within the AIHDA framework. For instance, Ian Hacking’s philosophy [46] and ecological niche metaphor [47] could help us understand how societal conditions shape the prevalence of certain issues [48] e.g., refraining from seeking PC.

For the study of PRs, we selected a reference stratum based on our *a priori* assumption that non-discriminated native men with high educational levels and aged 35–49 years should have the highest structural privileges in society. However, six strata of older individuals have a lower relative risk of refraining from seeking PC. The analysis based on the chosen reference reflects the relative intersectional inequalities, and we therefore decided to keep it.

Primary Health Care (PHC) clinics have an important role in reducing inequities in health by, *inter alia*, reaching and empowering individuals refraining from seeking medical care, so individuals can make decisions regarding their health and behaviour [49]. In the Swedish context, an earlier study suggested that refraining from seeking medical care due to perceived discrimination is associated with a relative lack of financial resources and previous negative experiences in the health sector [50]. This highlights that, although the place where the reported discrimination happened was not specified in this study, some social discrimination occurs in healthcare [51] and should be addressed to reduce barriers and increase access to healthcare.

Furthermore, structural barriers have been identified in Sweden when investigating the impact of the new PHC Choice reform passed in 2008. This reform aimed to allow all residents in Sweden to freely choose among PHC clinics. In addition, involving private providers was considered as a strategy for increasing access to healthcare. The reform has led to an increased number of (publicly financed) private PHC clinics as well as a higher number of healthcare visits. However, the benefits of the reform and its functioning, i.e., how PHC clinics respond to financial incentives and are governed, are being questioned as new clinics show reluctance to establish practices in areas with high levels of need for healthcare [52, 53]. This reform has likely increased inequalities in access to care by channeling resources to healthier people with more resources and power, instead of being needs-oriented on equal terms [54]. However, we need studies that adopt an intersectional AIHDA approach, to investigate the impact of the PHC reform on access to healthcare.

The challenge of addressing health inequities and discrimination extends beyond the healthcare sector, necessitating a multi-faceted approach. While healthcare plays a crucial role, it alone cannot resolve these complex issues [18]. It is thus imperative to promote control, influence, and possibilities to participate in society to improve health for all individuals [18]. The concept of social capital is often employed to assess the extent to which individuals have access to social networks and the degree of trust they place in both other individuals and the institutions within society. Social capital, a social and contextual factor, is believed to influence health through various mechanisms. These mechanisms encompass the shaping of health-related behaviours by prevailing norms and attitudes, improved access to healthcare by psychosocial networks, and the enhancement of self-esteem through psychosocial mechanisms [55, 56]. Thus, social capital might lead to better access to information, services and support which could lead to better health [55, 57]. More studies are needed in this area, particularly on how social capital is distributed across intersectional strata in the population and how social capital is related to discrimination [58].

As previously discussed [26, 42], comparing group averages is the *sine qua non* of epidemiology. However, by doing so, the same average risk value tends to be attributed to all individuals in the group, including those individuals placed in the tails of the within stratum risk distribution. Consequently, traditional epidemiological studies always convey some degree of unnecessary stigmatization of the individuals in “high-risk” groups whose individual predicted risk is low. Although, this could be ethically justified since the aim is to improve the health of the “high-risk groups”. Nevertheless, it is complex as stigmatization per se has negative social and medical consequences [59]. Therefore, the AIHDA approach has ethical value, as information on the DA of the grouping at hand may reduce the risk of unnecessary stigmatization [60, 61]. Analogously, comparing group averages may create false expectations of being protected from the outcome among the individuals in the “low-risk” groups whose individual predicted risk is high. Epidemiological “stigmatization” corresponds with the existence of false positive cases, while “false expectations” corresponds with false negative cases and justifies the relevance of using measures of DA. Epidemiologists, as well as medical and public health practitioners, frequently interpret the categories of exposure (e.g., of a risk factor, neighbourhoods, intersectional strata) as a kind of diagnostic test to identify individuals who will suffer from the outcome. As in any test, knowledge of its sensitivity and specificity is fundamental [31, 32, 41], which underlines the importance of the analysis of the AUC, for information on the DA.

In addition to the potential effects of stigmatization, overlooking measures of DA may lead to ineffective and inefficient interventions [42]. For instance, according to the idea of proportionate universalism for resource allocation in public health [62, 63], interventions mitigating health inequalities should be universal, i.e., directed to the whole population. However, universal interventions should also be combined with targeted actions proportionate to the level of disadvantage in specific population strata. The extent to which a universal intervention needs to be proportional can be evaluated by the DA of the intersectional strata [42, 64]. If the DA is large, it would be reasonable to focus on specific strata despite the risk of stigmatization of such groups. In our study, the DA of the intersectional information was small. Thus, many individuals refraining from seeking PC were in large strata with low average risks, i.e., false negatives. Therefore, restricting interventions to the high-risk strata would entail missing many individuals in the low-risk strata who, nevertheless, refrain from seeking PC. The low DA indicates the need for universal interventions that could, however, be tailored to the specific contextual characteristics of the different strata. This idea is similar to that of Rose’s epidemiological paradox [65], indicating that most cases may occur in the population without the risk factor (i.e., false negatives). Therefore, we need to consider a balance between population and high-risk strategies of prevention.

### Strengths and limitations

Our study has several strengths in addition to the advantages of the AIHDA approach discussed above. It is based on a large, pooled survey database assembled by The Public Health Agency of Sweden and representing the whole country. Information on refraining from physician's care is only available by using a survey. However, we excluded the surveys performed after 2014 as they had removed the question of refraining from PC.

This study could have been performed using multilevel models (i.e., MAIHDA). Although both the AIHDA and MAIHDA approaches conceptually consider the intersectional strata as contexts, MAIHDA provides methodological and conceptual advantages when it comes to the analysis of intersectional strata, compared to traditional regression analyses [26, 33, 42]. However, AIHDA is a useful and accessible alternative that shares crucial advantages with the MAIHDA approach, such as the provision of an intersectional mapping, and going beyond average probabilistic measurement and analysing the DA.

A limitation is the cross-sectional observational design of this study, which does not allow for the drawing of causal conclusions. AIHDA is mainly a descriptive analysis. However, the extended stratification allows for causal reasoning and ensures that, e.g., the association between discrimination and refraining from seeking PC is consistent across all the strata. Also, individuals with multiple social identities can experience various types of discriminatory treatment over their life course [19]. The change in form, intensity, and potential importance of the discrimination over the life course could not be investigated, which may lead to missed possibilities for prevention [66].

Additionally, the variables used in this study yield certain limitations. The outcome variable refraining from seeking PC is based on the individuals’ perceived need and this perception is subjective, and it may not be in concordance with a professional perspective of the need for care. Regarding the variable measuring discrimination, there are several aspects to consider. Firstly, perceptions of whether a certain treatment was discriminatory or not may differ between individuals depending on, for example, previous experiences, level of education, personality traits and the social context. Secondly, the assessment of the offensive or abusive treatment that induces feelings of humiliation may fail to encompass the full scope of the concept of ‘discrimination’ [67]. However, it has been demonstrated that perceived social discrimination contributes to health disparities and influences health status through delays in seeking healthcare [68, 69]. Therefore, wherever it occurs, social discrimination is a barrier to accessing healthcare that impairs individual health, which is in alignment with this study’s results showing discrimination being related to refraining from seeking medical care. Besides, it has been shown that perceived social discrimination reflects objective social discrimination [70]. Additionally, we only used five discrimination grounds as *“discrimination”* in this study. This, together with our exclusion of grounds of discrimination introduced in the survey in more recent years, such as skin colour and gender identity, might lead to an underestimation of the effects of discrimination in refraining from seeking medical care in this study’s results, as response options representing other grounds of discrimination (than, e.g., ethnicity or sex/gender) may have been chosen as the attributed reason for the discriminatory treatment. This entails a risk of bias in this study’s results. Furthermore, the available data does not exclusively pertain to discrimination within the healthcare sector, which limits our analysis. In light of this, further research with more specific measures of discrimination both generally and specifically in healthcare settings is needed to explore the specific impact of discrimination and avoided medical care in Sweden.

A further limitation of this study may lie in the limited number of dimensions and categorizations used to construct the intersectional strata. For instance, sexual orientation was not included, and sex was included as a binary variable neglecting the existence of other gender identities. Furthermore, educational level was defined in a binary manner (high/low) and individuals younger than 25 years were excluded for previously mentioned reasons. The categorizations used were based on the information available from the surveys but also to avoid strata containing few or no individuals. This restriction also applies to the use and categorization of country of birth as a binary variable (native/foreign-born) which may be acceptable but is rudimentary [71].

An additional limitation of this study pertains to the potential selection bias affecting the generalizability of our findings. An analysis of the non-responses conducted by Statistics Sweden shows that people of younger age, men, born outside Sweden, and with lower educational attainment responded to the questionnaire to a lower extent [72]. To correct imbalances between the sample and the general population, we used survey weights provided by Statistic Sweden, but the risk of selection bias remains for the groups that are not well represented among responders.

The discrepancies between the different language versions of the questionnaire and the possible variations in participants’ interpretations of the terms *“läkarvård”* vs “medical care” pose a potential source of bias in our study results. Specifically, those respondents who completed the questionnaire in English may have a distinct understanding of these terms compared to their counterparts who answered in Swedish. A potential divergence in interpretation might lead to inaccurate results, obscuring patterns of refrainment of physician-provided care.

## Conclusions

This study shows how the risk of refraining from seeking PC in Sweden is distributed across intersectional strata defined by combinations of categories of country of birth, age, sex, educational achievement and reported experiences of discrimination. From an individual perspective, having experienced discrimination and being foreign-born was associated with an increased average risk of refraining from seeking PC in most intersectional strata. However, from a population perspective, the DA of the intersectional strata was low so the risk of refraining from seeking PC was spread across the population. Therefore, potential interventions to eliminate discrimination and to improve access to medical care should be universally directed to the whole population rather than exclusively targeted to strata with the highest average risk, although tailored to fit the contexts of the intersectional strata. Our results underscore the importance of avoiding discrimination not least within healthcare, to increase access to healthcare, particularly among less privileged groups. It is also crucial to address structural discrimination and evaluate the reforms changing the healthcare system in Sweden, adapting it to the findings to improve needs-oriented care on equal terms.

## Supplementary Information

Supplementary Material 1.

## Data Availability

The dataset analysed during the current study is available from the Public Health Agency of Sweden on request.

## Abbreviations

AIHDA: Analysis of individual heterogeneity and discriminatory accuracy
AUC: Area under the ROC
AR: Absolute risk
ARD: Absolute risk difference
CI: Confidence intervals
DA: Discriminatory accuracy
MAIHDA: Multilevel analysis of individual heterogeneity and discriminatory accuracy
NPHS: National Public Health Surveys
PHA: Public Health Agency
PC: Physician’s care
PHC: Primary health care
PPV: Positive Predictive Value
PR: Prevalence Ratio
ROC: Receiver operating characteristics curve
TPR: Total Population Register (Swedish: *Registret **över **total befolkningen*)
