# Supplementary material for: Perceived discrimination and refraining from seeking physician’s care in Sweden: an intersectional analysis of individual heterogeneity and discriminatory accuracy (AIHDA)
Source: Int J Equity Health. 2024 Oct 5;23:199. doi: 10.1186/s12939-024-02291-4 (PMC11452949; doi:10.1186/s12939-024-02291-4)
Supplement: Supplementary file 1 — Supplementary Material 1. [file 12939_2024_2291_MOESM1_ESM.docx]

**Supplementary information (SI)**

| SI 1. Ranked prevalence ratios (PR) and 95% confidence intervals (CI) for all 64 intersectional strata obtained from the regression analysis in model 4 and number of individuals (cases) that refrain from seeking physician’s care in relation to the number of individuals in respective strata. | | | | | | | | |
| --- | --- | --- | --- | --- | --- | --- | --- | --- |
| Stratum rank | **Perceived discrimination** | **Country of birth** | **Sex** | **Education** | **Age** | **Number of cases** | **Number of individuals** | **PR (95% CI)** |
| 1 | No | Native | Man | High | 65–84 | 5208 | 90039 | 0.52 (0.42–0.65) |
| 2 | Yes | Foreign-born | Man | High | 65–84 | 59 | 890 | 0.60 (0.08–4.28) |
| 3 | No | Native | Man | High | 50–64 | 18767 | 216104 | 0.79 (0.67–0.92) |
| 4 | No | Native | Man | Low | 65–84 | 35360 | 404272 | 0.80 (0.69–0.91) |
| 5 | No | Native | Woman | High | 65–84 | 8829 | 98512 | 0.82 (0.68–0.98) |
| 6 | No | Native | Woman | Low | 65–84 | 44657 | 423195 | 0.96 (0.84–1.10) |
| 7 | No | Native | Man | High | 35–49 | 31420 | 285193 | *1.0 Reference* |
| 8 | No | Native | Woman | High | 50–64 | 27345 | 224343 | 1.11 (0.96–1.27) |
| 9 | No | Native | Man | Low | 50–64 | 70503 | 537921 | 1.19 (1.05–135) |
| 10 | No | Foreign-born | Man | High | 65–84 | 2184 | 16262 | 1.20 (0.80–1.79) |
| 11 | No | Native | Woman | High | 35–49 | 42307 | 307932 | 1.25 (1.09–1.42) |
| 12 | No | Native | Man | High | 25–34 | 28184 | 201429 | 1.27 (1.08–1.49) |
| 13 | No | Native | Woman | Low | 50–64 | 71998 | 483927 | 1.35 (1.19–1.53) |
| 14 | No | Foreign-born | Man | Low | 65–84 | 8451 | 55161 | 1.40 (1.12–1.77) |
| 15 | No | Foreign-born | Woman | High | 65–84 | 2456 | 15576 | 1.44 (1.01–2.05) |
| 16 | No | Native | Man | Low | 25–34 | 35390 | 220345 | 1.46 (1.25–1.70) |
| 17 | No | Native | Woman | High | 25–34 | 34542 | 209220 | 1.50 (1.30–1.72) |
| 18 | No | Native | Man | Low | 35–49 | 77136 | 465489 | 1.51 (1.32–1.71) |
| 19 | No | Foreign-born | Man | High | 25–34 | 5827 | 34594 | 1.54 (1.13–2.09) |
| 20 | No | Native | Woman | Low | 35–49 | 63800 | 368870 | 1.57 (1.38–1.78) |
| 21 | No | Foreign-born | Woman | Low | 65–84 | 11399 | 63668 | 1.63 (1.34–1.98) |
| 22 | Yes | Native | Man | High | 65–84 | 572 | 2725 | 1.87 (1.10–3.19) |
| 23 | No | Foreign-born | Man | High | 35–49 | 12980 | 62345 | 1.90 (1.54–2.33) |
| 24 | No | Foreign-born | Man | High | 50–64 | 8470 | 40072 | 1.92 (1.52–2.43) |
| 25 | No | Foreign-born | Woman | High | 50–64 | 9706 | 42758 | 2.05 (1.67–2.51) |
| 26 | No | Native | Woman | Low | 25–34 | 36941 | 162278 | 2.07 (1.80–2.39) |
| 27 | No | Foreign-born | Woman | High | 35–49 | 17025 | 73374 | 2.11 (1.77–2.53) |
| 28 | No | Foreign-born | Man | Low | 25–34 | 10075 | 38942 | 2.35 (1.84–3.01) |
| 29 | No | Foreign-born | Man | Low | 35–49 | 20582 | 79335 | 2.35 (1.96–2.83) |
| 30 | Yes | Native | Woman | High | 65–84 | 1398 | 5464 | 2.35 (1.68–3.29) |
| 31 | No | Foreign-born | Woman | High | 25–34 | 11603 | 44435 | 2.36 (1.92–2.91) |
| 32 | No | Foreign-born | Woman | Low | 25–34 | 9232 | 35289 | 2.37 (1.87–3.00) |
| 33 | No | Foreign-born | Man | Low | 50–64 | 22681 | 86919 | 2.39 (2.03–2.83) |
| 34 | No | Foreign-born | Woman | Low | 50–64 | 24209 | 91147 | 2.40 (2.05–2.82) |
| 35 | Yes | Foreign-born | Man | High | 25–34 | 1125 | 3983 | 2.47 (1.27–4.79) |
| 36 | No | Foreign-born | Woman | Low | 35–49 | 24251 | 85143 | 2.61 (2.21–3.07) |
| 37 | Yes | Native | Woman | High | 50–64 | 3292 | 11007 | 2.68 (2.09–3.43) |
| 38 | Yes | Native | Man | Low | 65–84 | 2626 | 8907 | 2.69 (2.00–3.60) |
| 39 | Yes | Native | Man | High | 50–64 | 1625 | 5449 | 2.77 (1.94–3.97) |
| 40 | Yes | Native | Man | High | 25–34 | 2058 | 6600 | 2.87 (1.92–4.31) |
| 41 | Yes | Native | Woman | High | 35–49 | 6466 | 19714 | 2.98 (2.42–3.67) |
| 42 | Yes | Native | Woman | Low | 50–64 | 6398 | 18838 | 3.12 (2.54–3.84) |
| 43 | Yes | Native | Man | High | 35–49 | 2211 | 6310 | 3.17 (2.21–4.53) |
| 44 | Yes | Foreign-born | Woman | High | 65–84 | 535 | 1516 | 3.18 (1.67–6.03) |
| 45 | Yes | Native | Woman | Low | 65–84 | 3517 | 10090 | 3.22 (2.52–4.13) |
| 46 | Yes | Native | Woman | High | 25–34 | 12342 | 34385 | 3.25 (2.74–3.86) |
| 47 | Yes | Native | Man | Low | 50–64 | 5996 | 16124 | 3.37 (2.70–4.22) |
| 48 | Yes | Native | Man | Low | 35–49 | 5104 | 13512 | 3.40 (2.61–4.44) |
| 49 | Yes | Foreign-born | Man | Low | 65–84 | 1145 | 3041 | 3.52 (2.21–5.61) |
| 50 | Yes | Native | Woman | Low | 35–49 | 8161 | 20655 | 3.59 (2.95–4.37) |
| 51 | Yes | Native | Woman | Low | 25–34 | 8771 | 21197 | 3.74 (3.07–4.55) |
| 52 | Yes | Foreign-born | Woman | Low | 25–34 | 2361 | 5735 | 3.93 (2.74–5.64) |
| 53 | Yes | Foreign-born | Man | High | 35–49 | 3350 | 7911 | 3.95 (2.89–5.38) |
| 54 | Yes | Foreign-born | Man | Low | 25–34 | 3619 | 8082 | 4.05 (2.86–5.73) |
| 55 | Yes | Foreign-born | Woman | High | 35–49 | 4562 | 10224 | 4.08 (3.14–5.30) |
| 56 | Yes | Foreign-born | Man | Low | 35–49 | 4734 | 10296 | 4.21 (3.13–5.65) |
| 57 | Yes | Foreign-born | Woman | Low | 50–64 | 3904 | 7617 | 4.63 (3.58–5.98) |
| 58 | Yes | Native | Man | Low | 25–34 | 7044 | 13064 | 4.90 (3.95–6.09) |
| 59 | Yes | Foreign-born | Man | High | 50–64 | 2988 | 5393 | 4.92 (3.64–6.66) |
| 60 | Yes | Foreign-born | Woman | High | 50–64 | 3812 | 6853 | 4.96 (3.90–6.31) |
| 61 | Yes | Foreign-born | Man | Low | 50–64 | 4558 | 8001 | 5.20 (4.10–6.59) |
| 62 | Yes | Foreign-born | Woman | High | 25–34 | 4560 | 7510 | 5.37 (4.25–6.78) |
| 63 | Yes | Foreign-born | Woman | Low | 65–84 | 3053 | 5071 | 5.45 (4.28–6.95) |
| 64 | Yes | Foreign-born | Woman | Low | 35–49 | 6954 | 10380 | 6.07 (5.05–7.30) |
